# MicroRNA-9 promotes tumor metastasis via repressing E-cadherin in esophageal squamous cell carcinoma

**DOI:** 10.18632/oncotarget.2581

**Published:** 2014-10-31

**Authors:** Ye Song, Jiangchao Li, Yinghui Zhu, Yongdong Dai, Tingting Zeng, Lulu Liu, Jianbiao Li, Hongbo Wang, Yanru Qin, Musheng Zeng, Xin-Yuan Guan, Yan Li

**Affiliations:** ^1^ State Key Laboratory of Oncology in South China and Collaborative Innovation Center for Cancer Medicine, Sun Yat-sen University Cancer Center, Guangzhou, 510060, China; ^2^ Vascular Biology Research Institute, Guangdong Pharmaceutical University, Guangzhou, 510060, China; ^3^ Department of Clinical Oncology, the First Affiliated Hospital, Zhengzhou University, Zhengzhou, 510060, China; ^4^ Department of Clinical Oncology, The University of Hong Kong, Hong Kong, China

**Keywords:** *miR-9*, ESCC, E-cadherin, metastasis, β-catenin

## Abstract

MicroRNAs (miRNAs) play a critical role in development and progression of cancers. Deregulation of MicroRNA-9 (*miR-9*) has been documented in many types of cancers but their role in the development of esophageal squamous cell carcinoma (ESCC) has not been studied. This study aimed to investigate the effect of miR-9 in esophageal cancer metastasis. The up-regulation of *miR-9* was frequently detected in primary ESCC tumor tissue, which was significantly associated with clinical progression (*P* = 0.022), lymph node metastasis (*P* = 0.007) and poor overall survival (*P* < 0.001). Functional study demonstrated that *miR-9* promoted cell migration and tumor metastasis, which were effectively inhibited when expression of *miR-9* was silenced. Moreover, we demonstrated that *miR-9* interacted with the 3′-untranslated region of *E-cadherin* and down-regulated its expression, which induced β-catenin nuclear translocation and subsequently up-regulated c-myc and CD44 expression. In addition, *miR-9* induced epithelial-mesenchymal transition (EMT) in ESCC, a key event in tumor metastasis. Taken together, our study demonstrates that *miR-9* plays an important role in ESCC metastasis by activating β-catenin pathway and inducing EMT via targeting E-cadherin. Our study also suggests *miR-9* can be served as a new independent prognostic marker and/or as a novel potential therapeutic target for ESCC.

## INTRODUCTION

Esophageal cancer is one of the most common solid malignancies in the world and ranks as the sixth-leading cause of cancer-related mortality [1]. Esophageal squamous cell carcinoma (ESCC) is the predominant histologic type in East Asia, especially in the high-risk areas in northern China [2, 3]. Despite the great advances achieved in diagnosis and multimodality therapies recently, the prognosis of ESCC is still poor with a dismal 5-year survival rate of 20–30% [4]. The high probability of metastasis and recurrence is still the major reason of grim prognosis, yet the precise molecular mechanism of metastatic dissemination is still not completely clear [3]. Therefore, understanding the factors involved in ESCC metastasis is required for the identification of new prognostic biomarkers and therapeutic targets.

In the last few years, growing body of evidences indicate that microRNAs (miRNAs) are involved in multiple cellular processes as posttranscriptional regulators and particularly in cancer development and progression [5, 6]. miRNAs are a diverse class of 20–24 nucleotide that plays important roles in gene regulation by pairing to the 3′-untranslated region (3′-UTR) of target mRNAs to direct their posttranscriptional repression [7]. Deregulation of miRNAs has been reported to play roles in ESCC metastasis by acting as activators or inhibitors [8–10]. In our recent study, microarray strategy was applied to identify differentially expressed miRNAs in ESCC cells by comparing miRNA profiles between tumor and paired non-tumor tissues [11]. *MiR-9* was investigated in this study because its deregulation has been reported in several types of cancers, including breast cancer [12], colorectal cancer [13] and melanoma [14]. However, the role of *miR-9* in the development and progress of ESCC remains unclear. In the present study, overexpression of *miR-9* was frequently detected in primary ESCC cases, which was associated with clinical progression, lymph node metastasis and poor overall survival. Functional study found that *miR-9* increased cell motility *in vitro* and tumor metastasis *in vivo*. Although E-cadherin has been documented as a target of *miR-9* in breast cancer [12], we further demonstrated that *miR-9* directly targeted the 3′-UTR of E-Cadherin and activated the β-catenin signaling pathway in ESCC.

## RESULTS

### Up-regulation of miR-9 is frequently detected in ESCC

**Figure 1:**
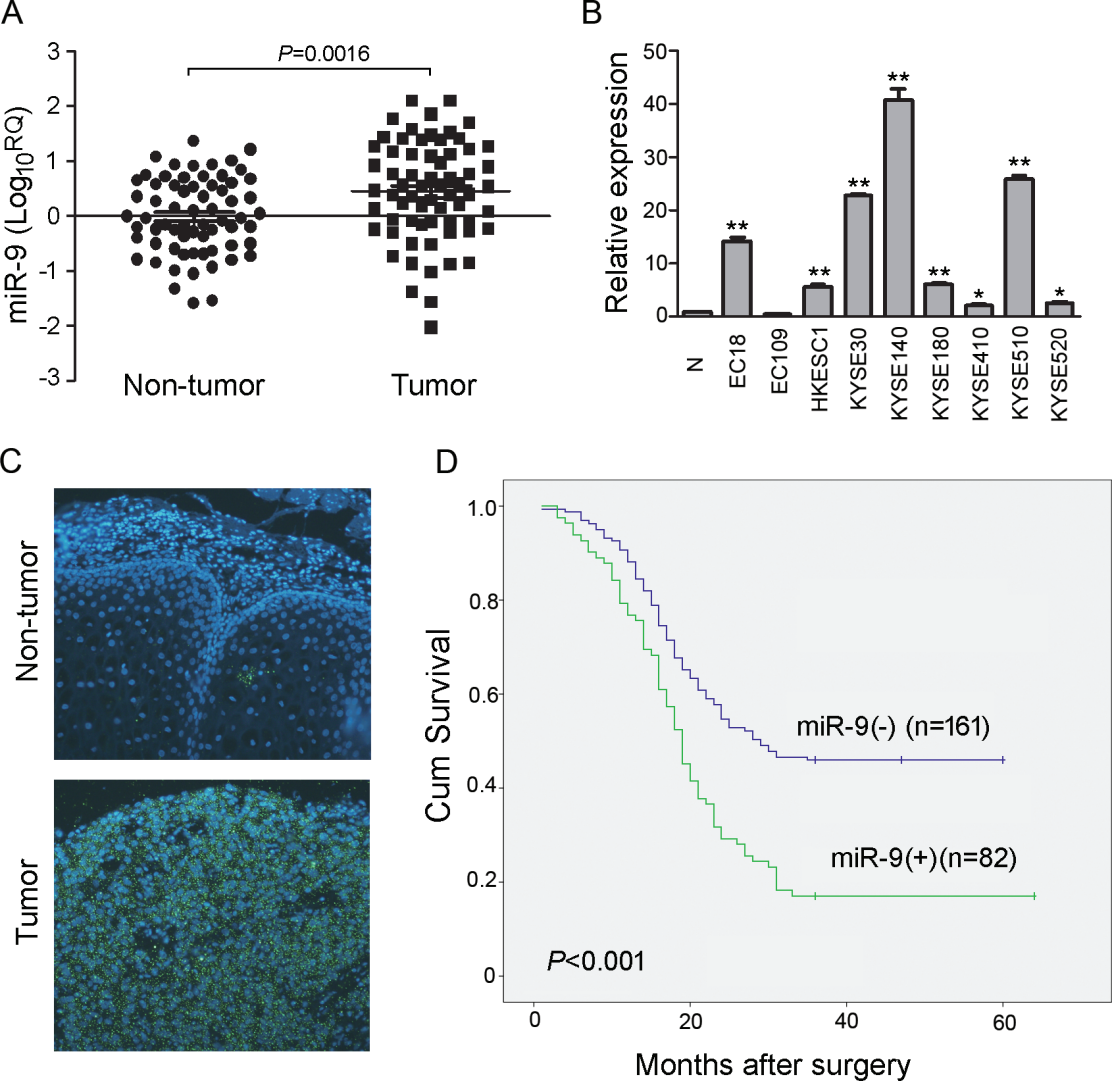
*miR-9* was frequently up-regulated in primary ESCC cases and cell lines **(A)** qRT-PCR shows that *miR-9* was frequently up-regulated in 67 primary ESCC tissues compared with their adjacent non-tumor tissues. (*P* = 0.0016, independent *t* test). Expression of miR-9 was shown in log_10_ scale and normalized to U6. **(B)** Up-regulation of *miR-9* was detected in all tested ESCC cell lines except EC109 compared with pool of non-tumor tissues (N). U6 was set as an endogenous control. *P < 0.05; **P < 0.001. **(C)** Representative, of *miR-9* expression (green signals) in a pair of ESCC tumor tissue and corresponding non-tumor tissue detected by MISH. Nuclei were conterstained by DAPI (blue color). Original magnification, 40 × objective. **(D)** Kaplan-Meier analysis indicates up-regulation of miR-9 was significantly associated with poorer overall survival rates of ESCC patients (*P* < 0.001).

To evaluate expression situation of *miR-9* in clinical ESCC specimens, quantitative real-time PCR (qRT-PCR) was used to compare expression levels of *miR-9* between tumor and corresponding non-tumor esophageal mucosa in 67 ESCCs. Up-regulation of *miR-9* was detected in 35/67 (52.24%) of ESCC tumors, compared with corresponding nontumorous tissues (defined as >2-fold increase). The average *miR-9* expression was significantly higher in tumor tissues than in their normal counterparts (*P =* 0.0016) (Figure 1A). Expression level of *miR-9* in 9 human ESCC cell lines was also detected by qRT-PCR and the result showed that up-regulation of *miR-9* could be detected in 8/9 cell lines (except EC109) compared with a pool of 5 nontumorous tissues as a normal control (Figure 1B).

### Up-regulation of miR-9 is associated with ESCC metastasis and poor prognosis

To investigate the clinical significance of *miR-9* up-regulation in ESCC, expression of *miR-9* was evaluated by microRNA *in situ* hybridization (MISH) in a tissue microarray containing 300 pairs of primary ESCCs and their paired non-tumor samples. Informative results were observed in 243 pairs of ESCC cases, while non-informative cases included lost samples and samples with limited number of cells. The overexpression of *miR-9*, defined as its fluorescent signals in tumor tissue obviously more and stronger than that in the corresponding non-tumor tissue, was detected in 82/243 (33.74%) of informative ESCC tissues (Figure 1C). The clinical association analysis found that overexpression of *miR-9* was significantly associated with advanced clinical stage (*P* = 0.022) and lymph node metastasis (*P* = 0.007, Table 1). Kaplan-Meier analysis indicated that overexpression of *miR-9* was significantly associated with poorer overall survival (log-rank test, *P* < 0.001, Figure 1D). Further, multivariate Cox regression analysis revealed that overexpression of *miR-9* is an independent prognostic factor for poor survival of patients with ESCC (*P* = 0.009, Table 2).

**Table 1:** Clinicopathological correlation of miR-9 expression in ESCC

Feature		miR-9 expression level		
	All	Normal	Upregulated	*P*
Age				0.069
≥60 years	139	98(70.5)	41(29.5)	
>60 years	104	63(60.6)	41(39.4)	
Differentiation				0.093
Well	60	33(55.0)	27(45.0)	
Moderate	156	108(69.2)	48(30.8)	
Poor	27	20(74.1)	7(25.9)	
Tumer invasion				0.705
T_1_	18	12(66.7)	6(33.3)	
T_2_	18	10(55.6)	8(44.4)	
T_3_	45	32(71.1)	13(28.9)	
T_4_	162	107(66.0)	55(34.0)	
Lymph node metastasis				0.007^*^
N_0_	138	101(73.2)	37(26.8)	
N_1_	105	60(57.1)	45(42.9)	
Clinical stage				0.022^*^
Stage I-II	161	115(71.4)	46(28.6)	
Stage III-IV	82	46(56.1)	36(43.9)	

* Significant difference

**Table 2:** Cox proportional hazard regression analyses for overall survival

Clinicopathological Features	Univariate Analysis		Multivariate Analysis	
	HR(95%Cl)	*P*	HR (95%Cl)	*P*
miR-9 UPregulation	1.913(1.391–2.632)	<0.001*	1.543 (1.112–2.140)	0.009*
Gender	0.785(0.568–1.085)	0.142		
Age	1.216(0.886–1.669)	0.225		
Differentiation	1.574(1.192–2.079)	<0.001*	1.465(1.114–1.928)	0.006*
Tumor invasion	1.481(1.197–1.831)	<0.001*	1.267(1.012–1.586)	0.039*
Clinical stage	2.483(1.802–3.420)	<0.001*	1.545 (0.799–2.990)	0.196
LN metastasis	2.138(1.556–2.938)	<0.001*	1.218 (0.654–2.268)	0.534

CI = confidence interval; HR = hazard ratio;

* Statistical significance (*p* < 0.05) is shown in bold.

### miR-9 promotes cell migration and tumor metastasis

**Figure 2:**
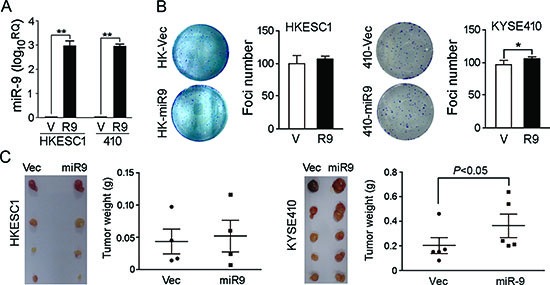
Functional study of *miR-9* **(A)** Relative expression of *miR-9* was detected by qRT-PCR in *miR-9*-transfected (R9) HKESC1 and KYSE410 cells compared with empty vector-transfected cells (V). **P < 0.001. **(B)** Foci formation assay was performed to compare frequency of foci formation between *miR-9*- and empty vector-transfected cells. Results are expressed as mean ±S.E.M. of three independent experiments. *P < 0.05. **(C)** Representative images of xenografts and summary of tumor weight in tumor formation in nude mice. *miR-9*- and empty vector-transfected cells were inoculated subcutaneously to the flanks of nude mice. Xenografts were isolated and weighted after 4 weeks.

**Figure 3:**
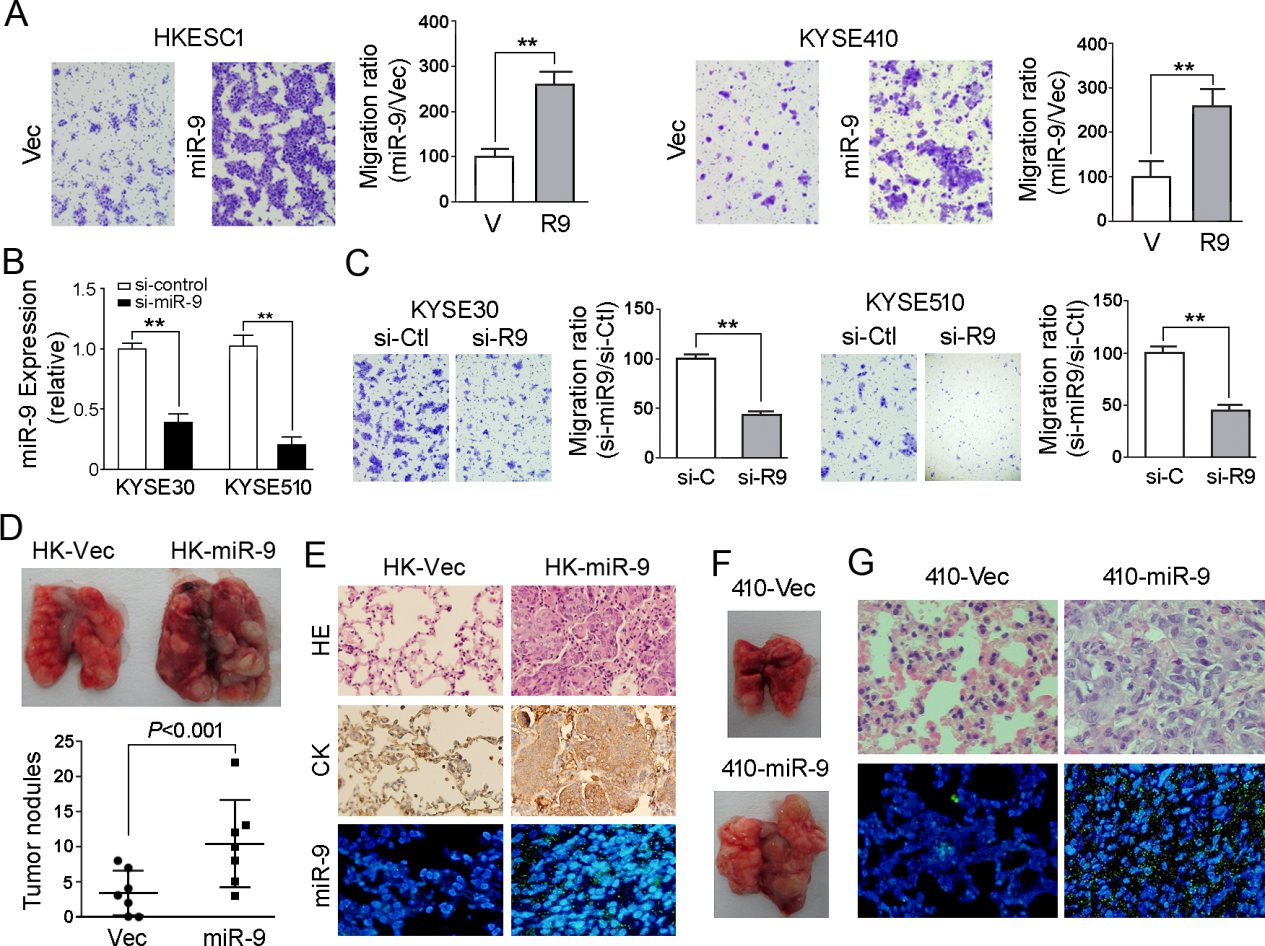
*miR-9* promotes cell migration and tumor metastasis **(A)** Representative images and summary of cell migration assay. Compared with empty vector-transfected cells, *miR-9*-transfected cells could promote cell migration in HKESC1 and KYSE410 cells. The results are expressed as mean ± S.E.M. of three independent experiments. **P < 0.01. **(B)** Down-regulation of *miR-9* was detected by qRT-PCR when *miR-9* was silenced by a siRNA in KYSE30 and KYSE510 cells. Cells treated with scramble siRNA were used as control cells. Results are expressed as mean ± S.E.M. of three independent experiments. **P < 0.01. **(C)** Representative images and summary of migrated cells between siRNA-*miR-9* and scramble siRNA treated KYSE30 and KYSE510 cells. The results are expressed as mean ± S.E.M. of three independent experiments. **P < 0.01. **(D)** *miR-9* could promote ESCC metastasis *in vivo*. Representative images of lungs derived from mice injected with HK-miR-9 and control cells (*upper*). Visible tumor nodules were counted and summarized (*bottom*). **(E)** H&E staining (*upper*) and IHC staining of CK (*middle*) was performed on pulmonary sections derived from mice. Original magnification: 20 × objective. Expression of *miR-9* (green signals) was detected by MISH in pulmonary sections (*bottom*). Original magnification: 40 × objective. **(F)** Representative image of lung derived from mice injected with 410-*miR-9* and control cells. **(G)** H&E staining (*upper*) was performed on pulmonary sections derived from mice injected with 410-*miR-9* and control cells. Original magnification: 40 × objective. Expression of *miR-9* was detected by MISH in pulmonary sections (*bottom*). Original magnification: 20 × objective.

To investigate the oncogenic function of *miR-9*, *miR-9* was cloned into a lentiviral vector and stably transfected into ESCC cell lines HKESC1 and KYSE410 (Figure 2A). Empty vector-transfected cells were used as controls. Tumorigenic effect of *miR-9* was studied by XTT cell growth assay, foci formation assay and tumor formation in nude mice. Unexpectedly, no significant difference was detected by XTT assay between *miR-9* transfected cells and control cells (data not shown). Foci formation and tumor formation in nude mice showed that *miR-9* could increase number of foci formed and promote tumor growth in tested mice in KYSE410 cells, but not in HKESC1 cells (Figure 2B and 2C). Since overexpression of *miR-9* has been significantly associated with ESCC metastasis, its role in cell migration and invasion was then investigated by both *in vitro* and *in vivo* assays. Cell migration assay showed that *miR-9* could significantly increase cell motility in HKESC1 and KYSE410 cells compared with the empty vector-transfected cells (*P* < 0.01, Figure 3A). When endogenous *miR-9* was silenced by a siRNA against *miR-9* in KYSE30 and KYSE510 cells (Figure 3B), the number of migrated cells decreased significantly compared with scramble siRNA-transfected control cells (*P* < 0.01, Figure 3C).

To further validate the effect of *miR-9* on tumor metastasis, *in vivo* metastasis assay was performed in nude mice. *miR-9* expressing HKESC1 (HK-*miR-9*) and KYSE410 (410-*miR-9*) cells were injected into nude mice via tail vein, respectively. Empty vector trasfected cells were used as controls (HK-Vec and 410-Vec). Eight weeks later, tested mice were sacrificed and the metastatic modules formed on the lungs and livers were counted. No visible metastatic nodule was observed in livers of mice except one HK-*miR-9* mouse (data not shown). Pulmonary metastatic nodules induced by HK-*miR-9* cells and HK-Vec cells were detected in 7/7 and 5/7 of tested mice, respectively. As shown in Figure 3D, the number of pulmonary metastatic nodules induced by HK-*miR-9* cells was significantly higher than that induced by control cells (*P* < 0.001, independent student's *t* test). Histological study with H&E stained paraffin block sections confirmed that the pulmonary nodules were metastatic cancers (Figure 3E). IHC staining with anti-cytokeratin antibody was performed to validate that the cells originated from injected tumor cells (Figure 3E). MISH demonstrated that *miR-9* was overexpressed in metastatic nodule induced by miR-9 overexpressed cells compared with control cells (Figure 3E). The pulmonary metastatic nodules induced by 410-*miR-9* cells could be only observed in one mouse whereas no visible tumor nodule was observed in 410-Vec mice (Figure 3F). Histological and MISH studies confirmed the metastatic nodule was cancer with miR-9 overexpression (Figure 3G).

### E-cadherin is a putative target of miR-9 in ESCC

**Figure 4:**
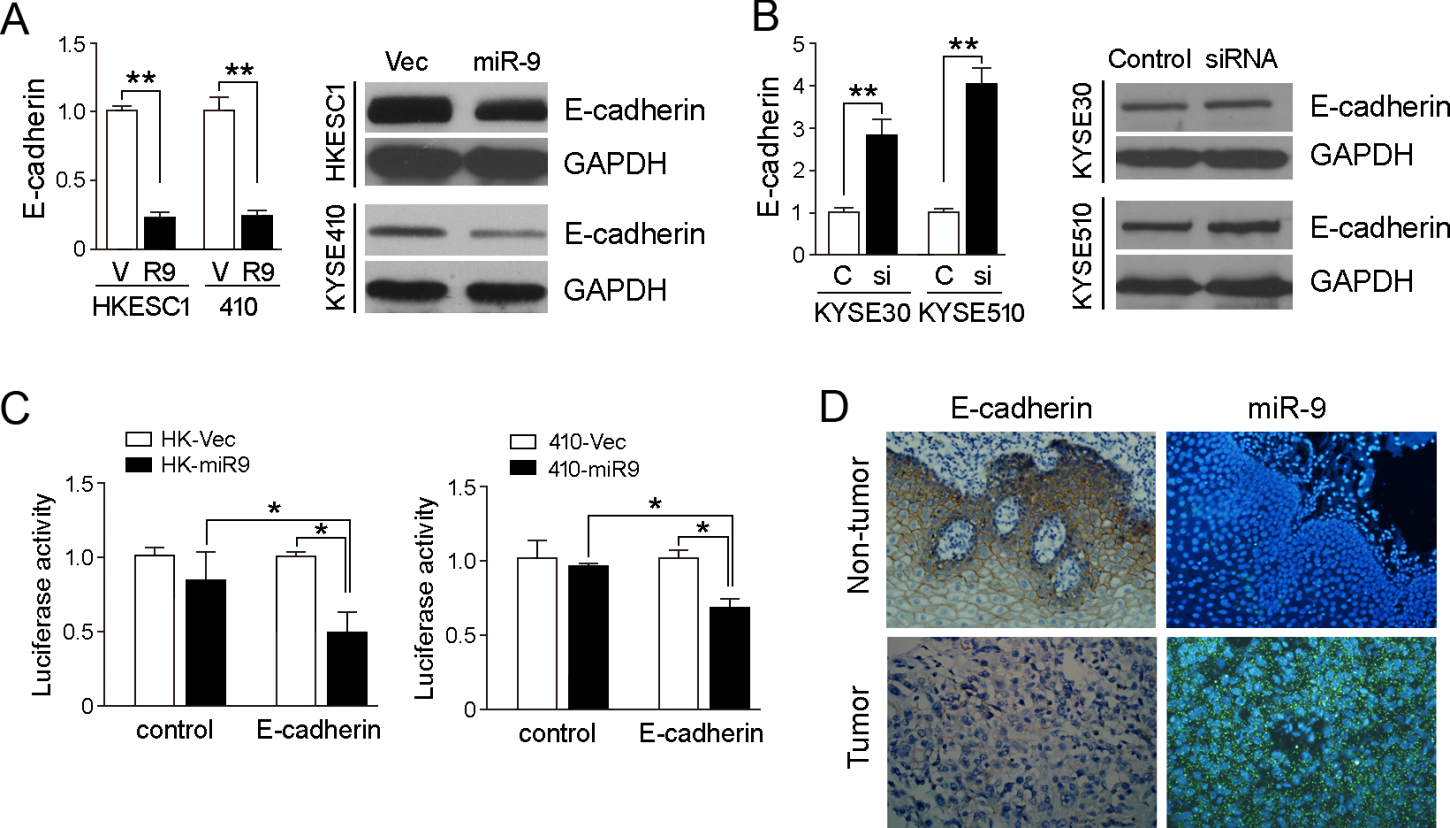
*miR-9* down-regulates E-cadherin in ESCC cells **(A)** qRT-PCR and Western blotting results showed that *miR-9* could effectively down-regulate E-cadherin expression in HK-*miR-9* and 410-*miR-9* cells compared with control cells. qRT-PCR results were expressed as mean ± S.E.M. of three independent experiments. **P < 0.001. GAPDH was set as an internal control in Western blot analysis. **(B)** Expressions of E-cadherin in both mRNA and protein levels were increased when *miR-9* was silenced in KYSE30 and KYSE510 cells by a siRNA against *miR-9*, compared with scramble siRNA-treated cells. C, control scramble siRNA; si, siRNA against *miR-9*. **P < 0.01. **(C)** Luciferase assay was performed to confirm that *miR-9* could target E-cadherin. *miR-9* overexpressed cells and control cells were co-transfected with pMIR-REPORT-CDH1(3′-UTR)/empty vector with pRL-TK, and relative luciferase activity was detected. *P < 0.05. **(D)** Representative pictures of E-cadherin staining (by IHC) and miR-9 (by MISH) in a pair of ESCC tumor tissue and corresponding non-tumor tissue. Results showed that E-cadherin was down-regulated in *miR-9* overexpressed tumor tissue. Original magnification: 20 × objective.

By searching the targets of *miR-9* with TargetScan and miRanda, one potential target E-cadherin was selected for further study because it has been associated with tumor metastasis [15]. 3^′^-UTR of E-cadherin mRNA contains one complementary site for *miR-9*. To examine whether E-cadherin is regulated by *miR-9* in ESCC, we tested the expression of E-cadherin in *miR-9* overexpressed cell lines HKESC1 and KYSE410. qRT-PCR and western blotting results showed that expression of E-cadherin was significantly reduced in HKESC1 and KYSE410 when *miR-9* was stably transfected into these cells compared with empty vector-transfected control cells (*P* < 0.01, Figure 4A). When *miR-9* was silenced in KYSE30 and KYSE510 cells, expression of E-cadherin was increased significantly (*P* < 0.01, Figure 4B).

A luciferase reporter assay was then performed to verify whether E-cadherin is the direct target of *miR-9*. The potential *miR-9* binding sequence in the 3′-UTR region of E-cadherin was cloned into a luciferase reporter vector and transiently transfected into the *miR-9* overexpressed cells and vector control cells. Decreased luciferase activity in *miR-9* overexpressed cells was observed compared with the control cell (Figure 4C), indicating that E-cadherin was a downstream target of *miR-9*.

### miR-9 level is negatively correlated with expression of E-cadherin in ESCC

To investigate the correlation between expressions of *miR-9* and E-cadherin in clinical samples, IHC was performed using a TMA containing 300 pairs of primary ESCCs. Informative results were observed in 237 pairs (both tumor and non-tumor tissues) of ESCCs. Non-informative cases were mainly caused by tissue loss in either tumor or paired non-tumor tissues. E-cadherin staining was calculated by adding the scores for the percentage and intensity of positive staining of E-cadherin. Since the staining index of E-cadherin in non-tumor samples was ≥5, down-regulation of E-cadherin was defined as staining index <5. Down-regulation of E-cadherin was detected in 103/237 (43.5%) of informative ESCC tissues compared with their paired non-tumor tissues (Figure 4D). Moreover, the correlation between *miR-9* and E-cadherin was analyzed by Pearson χ2 test in 216 cases with both miR-9 and E-cadherin information, a negative correlation was detected between *miR-9* and E-cadherin (*P* = 0.02, Supplementary Table 1).

### miR-9 promotes tumor metastasis via nuclear translocation of β-catenin

**Figure 5:**
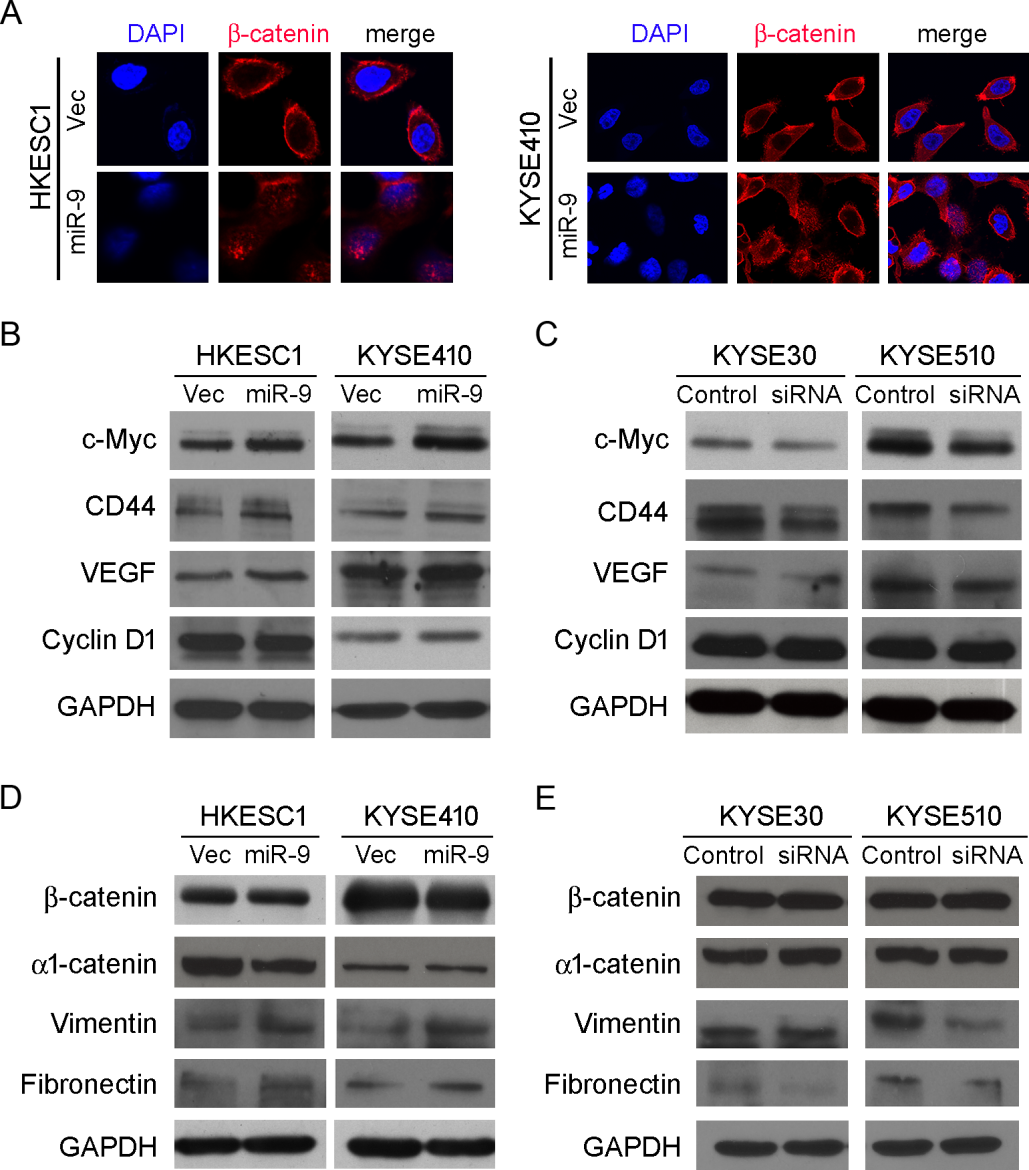
miR-9 activates β-catenin pathway and induces EMT **(A)** IF showed that nuclear translocation of β-catenin (red) happened in miR-9-transfected cells while β-catenin located mainly on the membrane in empty vector-transfected cells. Nuclei were counterstained with DAPI. Original magnification: 40 × objective. **(B-E)** Western blot analysis was used to compare protein expression levels between miR-9- and empty vector-transfected cells (B and D), or between cells treated with siRNA against *miR-9* and scramble siRNA (C and E). GAPDH was set as internal control.

E-cadherin/β-catenin complex is known for connecting the actin cytoskeleton to the adherens junctions [16]. When the cytoplasmic E-cadherin decreased, β-catenin is released from the complex. Stabilized “free” β-catenin goes to the nucleus, binds T-cell factor/lymphoid enhancer factor (TCF/Lef) and activates transcription of target genes such as c-myc, cyclin D1, CD44 and VEGF, which are responsible for cell proliferation and metastasis [17]. To explore the molecular mechanism of *miR-9*/E-cadherin in promoting metastasis, subcellular localization of β-catenin was detected by immunofluorescence (IF) analysis. IF result showed that β-catenin translocation from the cell membrane to nucleus was observed in both *miR-9*-transfected HKESC1 and KYSE410 cells, whereas β-catenin was mainly located on the membrane in the empty vector-transfected cells (Figure 5A). To determine whether the nuclear translocation of β-catenin can upregulate its downstream target genes, western blot analysis was used to test expressions of c-myc, cyclin D1, CD44 and VEGF. Increased expressions of c-myc, CD44 and VEGF were detected in HK-*miR-9* and 410-*miR-9* cells compared with their control cells (Figure 5B). No significant change was observed on cyclin D1 protein level. When *miR-9* was knocked down in KYSE30 and KYSE510 cells, the protein levels of c-myc, CD44 and VEGF were decreased (Figure 5C).

### miR-9 induces epithelial-mesenchymal transition in ESCC

As epithelial-mesenchymal transition (EMT) is one of the key events involved in invasion and metastasis of tumor cells, several epithelial markers (β-catenin and α_1_-catenin) and mesenchymal markers (Vimentin and Fibronectin) were tested by Western blot analysis. The results found that down-regulations of epithelial markers were not obvious except decrease of α_1_-catenin in HK-*miR-9* cells (Figure 5D). Upregulation of Vimentin and Fibronectin could be detected in both HK-*miR-9* and 410-*miR-9* cells (Figure 5D). In *miR-9* silenced KYSE30 and KYSE510 cells, mesenchymal markers were down-regulated except Vimentin in KYSE30 cells (Figure 5E). These data suggest that *miR-9* could induce partial EMT that promoted cancer cell invasion and metastasis.

## DISCUSSION

Previous studies have reported that miRNAs, such as oncomiRs or anti-oncomiRs, play important roles in cancer development and progression by acting as activators or inhibitors [18]. More recently, several specialized miRNAs termed metastamirs have been implicated in the regulation of tumor metastasis [19]. Metastamirs always play important roles in various steps of metastasis rather than tumorigenesis.[12, 19, 20] Characterization of metastamirs and their targets involved in the progression of ESCC may lead to the identification of novel prognostic markers and therapeutic targets. *miR-9*, a highly conserved microRNA, was reported to be expressed predominantly in the central nervous system of the developing embryo exhibiting a prodifferentiation function [21]. In addition to its involvement in neurogenesis, *miR-9* was first found to be up-regulated in primary brain tumors [22]. Up-regulation of *miR-9* has been also reported in breast cancer [12], colorectal cancer [13] and melanoma [14]. Interestingly, down-regulation of *miR-9* has been reported in gastric [23] and ovarian cancers [24], suggesting the diversity role of *miR-9* in different cancers.

Here we report that *miR-9* was frequently up-regulated in primary ESCC tumors, which was significantly associated with advanced clinical stage (*P* = 0.022), lymph node metastasis (*P* = 0.007) and poor survival rate (*P* < 0.001) of ESCC. Moreover, multivariate Cox regression analysis revealed that up-regulation of *miR-9* is an independent prognostic factor for poor survival of patients with ESCC. Functional study found that *miR-9* could increase cell migration and metastasis, implying that *miR-9* is a metastamiR in ESCC, and it could be used as a prognostic biomarker in ESCC.

As reported by many studies, informatics analysis indicated that E-cadherin might be the downstream target of *miR-9* [12, 25]. In the present study, E-cadherin was down-regulated in miR-9 overexpressed cells and up-regulated when endogenous *miR-9* was silenced. Luciferase assay also confirmed that *miR-9* could target E-cadherin directly. E-cadherin is a crucial protein mediated cell-cell adhesion to hold the epithelial cells tight together. Loss of E-cadherin decreases the cellular adhesion, resulting in an increase in cellular motility [26]. It has been reported that down-regulation of E-cadherin in esophageal carcinoma was associated with an increased invasive and metastatic potential [15]. Moreover, E-cadherin can sequester β-catenin as a complex to connect the actin cytoskeleton to the adherent junctions [26]. Upon loss of E-cadherin expression, β-catenin was released from the complex. Stabilized “free” β-catenin goes to the nucleus and modulates b-catenin/Tcf-mediated gene expression to promote cell invasion and metastasis [27]. It was also reported that loss of E-cadherin activates EGFR-MEK/ERK signaling and promotes invasion in non-small cell lung cancer [28]. Recently, Ma et al. reported that *miR-9* could target E-cadherin and allow liberation of β-catenin, which then activates the VEGF to promote metastasis in breast cancer [12]. Our results confirmed *miR-9* to be involved in regulation of cell metastasis by targeting E-cadherin in ESCC. In the ESCC primary tumor tissues, *miR-9* is negatively correlated with the expression of E-cadherin (R = −0.163, *P* < 0.05). Although *miR-9* could not cause significant change on β-catenin protein level, the nuclear translocation of β-catenin was observed in *miR-9* overexpressed cells. Western blotting analysis demonstrated that the downstream targets of β-catenin, including VEGF, c-myc and CD44 were up-regulated in *miR-9* overexpressed cells, suggesting that these genes may participate in the tumor metastasis in ESCC.

Since epithelial-mesenchymal transition is one of the key events involved in tumor invasion and metastasis [27], the effect of *miR-9* on EMT was also investigated in the study. As we mentioned earlier, no significant change on β-catenin protein level was observed. α_1_-catenin decreased in HK-*miR-9* cells and increased in *miR-9* silenced KYSE30 cells. Changes of 2 mesenchymal markers (Vimentin and Fibronectin) were also detected in *miR-9* overexpressed and silenced cells, suggesting that the metastatic effect of *miR-9* was via inducing EMT in ESCC. In summary, *miR-9* was frequently overexpressed in ESCC specimens and played a crucial role in ESCC metastasis through targeting E-cadherin, promoting β-catenin nuclear translocation and subsequently inducing EMT.

## MATERIALS AND METHODS

### Cell lines and primary tumor tissues

Six ESCC cell lines (KYSE30, KYSE140, KYSE180, KYSE410, KYSE510, and KYSE520) were obtained from DSMZ (Braunschweig, Germany), the German Resource Centre for Biological Material [29]. Three Chinese ESCC cell lines (EC18, EC109, and HKESC1) were kindly provided by our fellows at the University of Hong Kong (Professors G. Srivastava and G.S. Tsao) [30]. Sixty seven pairs of primary ESCCs and corresponding nontumorous tissues were collected directly after surgical resection at Linzhou Cancer Hospital (Henan, China). A total of 300 formalin-fixed and paraffin-embedded ESCCs and their adjacent nontumorous tissue samples were also kindly provided by Linzhou Cancer Hospital. All patients recruited in the study did not receive preoperative treatment. Clinical samples used in this study were approved by the Committees for Ethical Review of Research at Zhengzhou University (Zhengzhou, China) and Sun Yat-Sen University Cancer Center (Guangzhou, China).

### RNA isolation and quantitative real-time PCR

Total RNA was extracted with TRIzol (Invitrogen, Calsbad, CA) according to the manufacturer's instructions. Reverse transcription was performed using the PrimeScript^RT^ reagent Kit (Promega, Madison, WI). Real-time PCR was carried out using SYBR Green SuperMix (Roche, Basel, Switzerland) and ABI7900HT Fast Real-Time PCR system (Applied Biosystems, Foster City, CA). Glyceraldehyde-3-phosphate dehydrogenase (GAPDH) or U6 was used as internal control. The primers of miR-9 and U6 were purchased from HAPK Biotechnonology (Shenzhen, China). Other primer sequences used in the study were listed in supporting data.

### Tissue microarray (TMA) and immunohistochemical staining (IHC)

Tissue microarray containing 300 pairs of primary ESCC cases (tumor and corresponding non-tumor tissues) was constructed as described previously [31]. None of the patients in the study has received follow-up radiation or chemotherapy. IHC was performed using a standard streptavidin-biotin-peroxidase complex method. In brief, a TMA section was deparaffinized and rehydrated. Endogenous peroxidase activity was blocked with 0.3% hydrogen peroxide for 15 minutes. For antigen retrieval, the TMA slide was microwave-treated in 10 mM citrate buffer (pH 6.0) for 10 minutes. The slide was then incubated with anti-E-cadherin monoclonal antibody (Cell Signalling Technology, Danvers, MA). The nucleus was counterstained using Meyer's hematoxylin. E-cadherin immunoreactivity was calculated by adding the scores for the percentage of E-cadherin -positive cells (<5%, 0; 5%–25%, 1; 25%–50%, 2; 50%–75%, 3; >75%, 4) and the intensity of E-cadherin-positive staining (negative, 0; weak, 1; moderate, 2; or strong, 3). Informative results were observed in 237 pairs of ESCC cases, while non-informative samples included lost samples and inappropriate staining.

### microRNA in situ hybridization (MISH)

An oligonucleotide probe (5′-TCATACAGCTAGATAACCAAAGA-3′) complementary to hsa-miR-9-5p was purchased from the Exonbio Lab (Guangzhou, China). This oligonucleotide contains three 2′-fluoro-modified RNA residues (2′-F RNA) at the 4^th^, 10^th^ and 20^th^ bases, which can increase melting temperature and result in enhanced hybridization stability. Both 5′ and 3′ ends were labeled by digoxin (DIG). A scramble probe 5′-TCTTACACCTAGATAAGCAAAGA-3′ was used as a negative control. MISH was performed as described previously [32]. Informative results were observed in 243 pairs of ESCCs. Non-informative cases included lost samples and samples with limited number of cells.

### Establishment of miR-9 overexpression ESCC cell lines

Lentiviral construct containing pre-miR-9 (GeneCopoeia, China) was packaged using the ViraPower Mix (Invitrogen, Carlsbad, CA) in 293FT cells. pre-miR-9-expressing lentivirus was used to stably transfect ESCC cells (KYSE410 and HKESC1) to establish miR-9-overexpressing cells. Empty vector-transfected cells were established as controls. A siRNA against miR-9 (Genepharma, China) was transfected into KYSE30 and KYSE510 cells using Lipofectamine 2000 (Invitrogen, Carlsbad, CA) according to the manufacturer's instructions. The cells transfected with scrambled inhibitor (NC) (Genepharma, China) were used as negative controls.

### Cell growth assay, foci formation assay and in vivo tumor formation assay

For cell growth assay, 1 × 10^3^ cells were plated in 96-well plates and the cell growth rate was assayed by CCK-8 kit (Dojindo, Japan) according to the manufacturer's instruction. For foci formation assay, 1 × 10^3^ cells were plated in 6-well plates, After 2 weeks culture, colonies consisted of >50 cells were counted with 1% crystal violet staining. Triplicate independent experiments were performed.

Animal experiments were performed in compliance with the guidelines for the Welfare of Experimental Animals in Sun Yat-sen University Cancer Center. For *in-vivo* study, HK-*miR-9* (2 × 10^6^), 410-*miR-9* (3 × 10^6^) and empty vector transfected control cells were subcutaneously injected into the dorsal flanks of nude mice, respectively (4 for HKESC1 group and 5 for KYSE410 group). After 4 weeks, tumor formation in nude mice was examined by measuring tumor weight.

### Cell motility assay

Migration assay was performed with migration chamber (BD Biosciences, Flanklin Lakes, NJ) following the manufacturer's instructions. Cells located on the lower side of the chamber were fixed, stained, and counted under a microscope. Triplicate independent experiments were performed.

### *In vivo* metastasis assay

For the *in vivo* metastasis assays, 7 × 10^5^ cells were injected intravenously through the tail vein into 4- to 5-week-old nude mouse (7 for HKESC1 group and 6 for KYSE410 group), respectively. The mice were sacrificed 8 weeks later and the intrahepatic and pulmonary metastatic nodules were carefully examined and counted. The livers and lungs were fixed for further study. The tissue blocks were cut into 5 μm sections and stained with haematoxylin-eosin.

### Antibodies and Western blotting

Quantified protein lysates were resolved on SDS-PAGE gel, transferred onto PVDF membrane (Millipore, Billerica, MA), and immunoblotted with anti-human antibodies E-cadherin, β-catenin, Vimentin, α1-catenin, cyclin D1, c-myc (Cell Signalling Technology, Danvers, MA), GAPDH and CD44 (Santa Cruz, CA), VEGF (BD Biosciences, Franklin Lakes, NJ), and cytokeratin (Maixin, China). Blots were visualized with enhanced chemiluminescence (Amersham Biosciences, Piscataway, NJ).

### Luciferase assay

An 819-bp fragment of CDH1 3′-UTR containing the putative binding site of miR-9 was amplified by PCR (Supporting data) and cloned into pMIR-REPORT vector (Ambion, Carlsbad, CA). The miR-9 overexpressed cells and the vector control cells were transfected with the reporter constructs containing the target binding sequence of CDH1, the pMIR-REPORT vector was used as control. Renilla luciferase vector (pRL-TK) was cotransfected into KYSE410 and HKESC1 cells as a normalizing transfection control, Dual luciferase signals were measured 48 hours later by the Dual-luciferase assay kit (Promega, Madison, WI).

### Immunofluorescence (IF)

Immunofluorescence analysis was performed as described previously [33]. Briefly, cells were fixed and incubated with the primary antibody β-catenin (Immunofluorescence Preferred; Cell Signaling Technology, Danvers, MA) overnight at 4°C. After washing with PBS, cells were then incubated with fluorescence-conjugated secondary antibody (Invitrogen, Calsbad, CA). Images were captured after stained with Anti-fade DAPI solution.

### Statistical analysis

SPSS standard V.16.0 (SPSS, Inc., IL) was used for Statistical analysis, Student's t tests was used to analysis the significance of difference. Survival analysis was performed using Kaplan-Meier plots and log-rank tests. The correlation between miR-9 expression and clinicopathological characteristics was analyzed by Pearson χ2 test. Univariable and multivariable Cox proportional hazard regression model was used to assess the survival hazard. Differences were considered significant when *P* < 0.05.

## SUPPLEMENTARY DATA AND TABLE


