# Supplementary material for: MicroRNA-9 promotes tumor metastasis via repressing E-cadherin in esophageal squamous cell carcinoma
Source: Oncotarget. 2014 Oct 31;5(22):11669–80. doi: 10.18632/oncotarget.2581 (PMC4294333; doi:10.18632/oncotarget.2581)
Supplement: Supplementary file 1 [file oncotarget-05-11669-s001.pdf]

SUPPLEMENTARY DATA AND TABLE

Primers and sequences

**Primers:**	
E-cadherin	Forward: TGCCCAGAAAATGAAAAAGG
	Reverse: GTGTATGTGGCAATGCGTTC
GAPDH	Forward: CATGAGAAGTATGACAACAGCCT
	Reverse: AGTCCTTCCACGATACCAAAGT
**Cloning primers for luciferase assay:**	Forward: TCATACTAGTAAGTGATCCTCCCATCTTGG
	Reverse: GAATAAGCTTCAAACCACGGATCTTGTGTC
**predicted miR-9 binding sequence:**	
CDH1 3′ UTR	GCCUAAAGUGCUGCAGCCAAAGAC
miR-9	AGUAUGUCGAUCUAUUGGUUUCU

An 819-bp fragment of CDH1 3′-UTR containing the putative binding site of miR-9 cloned into pMIR-REPORT vector

aagtgatcctcccatcttggcctcccagagtattgggattacagacatgagccactgcacctgcccagctccccaactccctgccattttttaagagacagtttcgctccatcgcccaggcctgggatgcagtgatgtgatcatagctcactgtaacctcaaactctggggctcaagcagttctcccaccagcctccttttttattttttgtacagatggggtcttgctatgttgcccaagctggtcttaaactcctggcctcaagcaatccttctgccttggccccccaaagtgctgggattgtgggcatgagctgctgtgcccagcctccatgttttaatatcaactctcactcctgaattcagttgctttgcccaagataggagttctctgatgcagaaattattgggctcttttagggtaagaagtttgtgtctttgtctggccacatcttgactaggtattgtctactctgaagacctttaatggcttccctctttcatctcctgagtatgtaacttgcaatgggcagctatccagtgacttgttctgagtaagtgtgttcattaatgtttatttagctctgaagcaagagtgatatactccaggacttagaatagt*gcctaaagtgctgcagccaaaga*cagagcggaactatgaaaagtgggcttggagatggcaggagagcttgtcattgagcctggcaatttagcaaactgatgctgaggatgattgaggtgggtctacctcatctctgaaaattctggaaggaatggaggagtctcaacatgtgtttctgacacaagatccgtggtttg

Supplementary Table S1. Crosstable of miR-9 expression and protein expression of E-cadherin

		miR-9 expression				
		**Low, No (%)**	**High, No (%)**	**Total**	**R**	***P***
**E-cadherin by IHC**	Low expression	52(57.1)	39(42.9)	91	-0.163	*0.02*
	High expression	91(72.8)	34(27.2)	125		
